# Supplementary material for: WordCommentsAnalyzer: A windows software tool for qualitative research
Source: F1000Res. 2018 Sep 4;7:536. Originally published 2018 May 3. [Version 2] doi: 10.12688/f1000research.14819.2 (PMC6137411; doi:10.12688/f1000research.14819.2)
Supplement: Supplementary file 3 [file f1000research-7-17614-s0002.tgz › b2a79496-4ada-47c4-9682-a99ce867836d.zip!/Eight Iranian Health Journals/Journal of Research in Rehabilitation Sciences _47.docx]

© Journal of Research in Rehabilitation Sciences . This work is licensed under CC BY-NC 4.0 (https://creativecommons.org/licenses/by-nc/4.0/).

Volume & Issue: دوره 6، شماره 1: 1389

Title: مقایسه نتایج درمان غیر جراحی شکستگی‎های پایدار خارج مفصلی دیستال رادیوس به دو روش بی‎حسی موضعی و بی‎هوشی عمومی

http://jrrs.mui.ac.ir/index.php/jrrs/article/view/115

چکیده مقدمه: شکستگی کالیس (Colles) یکی از شایع‎ترین شکستگی‎های اندام در افراد بالغ است، زمانی که این شکستگی با ثبات باشد، درمان استاندارد شامل جااندازی بسته تحت بی‎حسی موضعی و یا بی‎حسی موضعی و یا بی‎هوشی عمومی و سپس گچ‎گیری، می‎باشد. این مطالعه به منظور مقایسه نتایج درمان غیر جراحی شکستگی کالیس تحت بی‎حسی موضعی و بی‎هوشی عمومی انجام گرفت. مواد و روش‌ها: در این کارآزمایی بالینی که به روش مداخله‎ای تصادفی انجام پذیرفت، 100 بیمار با شکستگی کالیس پایدار از مهرماه 85 لغایت مهرماه87 در مرکز درمانی کاشانی شهرستان اصفهان وارد مطالعه شدند. بیماران به طور تصادفی ساده به دو گروه مساوی الف و ب تقسیم گردیدند. در گروه الف، بیماران تحت درمان بی‎حسی موضعی و در گروه ب تحت بی‎هوشی عمومی قرار گرفتند. در پایان اطلاعات مربوط به نوع شکستگی، عوارض جااندازی، هزینه درمان و نتایج درمان در هفته‎های اول، دوم و سوم بعد از شروع درمان و سپس انتهای درمان بررسی شد. آن گاه نتایج به دست آمده از این 2 روش قبل و بعد از درمان با یکدیگر مقایسه شدند. یافته‌ها: آنالیز داده‎ها هیچ تفاوت معنی‎داری را بین عوارض این دو روش درمانی نشان نداد. علاوه بر این، هیچ تفاوت معنی‎داری بین معیارهای جااندازی 2 گروه یافت نشد، بین جنس و شکستگی کالیس نیز ارتباط مشخصی به دست آمد. آنالیز داده‎ها نیز نشان داد که هزینه درمان در گروه ب، 3 الی 4 برابر بیشتر از گروه الف می‎باشد. بحث: درمان جااندازی به روش بی‎حسی موضعی با توجه به هزینه کمتر و جااندازی قابل قبول بدون عارضه مشخص، بهتر از جااندازی به روش بی‎هوشی عمومی بود. پژوهشگران این مطالعه، استفاده از بی‎حسی موضعی جهت جااندازی شکستگی کالیس را توصیه کردند.

Title: ارزیابی وضعيت سر در دندان‎پزشكان فاقد درد گردن

http://jrrs.mui.ac.ir/index.php/jrrs/article/view/116

چکیده مقدمه: دندان‌پزشكي يكي از مشاغلي است كه به تمركز و دقت بالايي نياز دارد. فلكشن سر به مدت طولاني، ابداكشن بازوها، انجام عمليات درماني در فضاي كوچك دهان بيمار و پوسچر (وضعیت) شغلی غير قابل انعطاف از جمله فاكتورهايي هستند كه دندان‌پزشكان را مستعد اختلالات اسکلتی- عضلانی می‌‌سازد. با این حال، به وجود آمدن وضعیت غیر طبیعی رو به جلوی سر، پیش از آغاز گردن درد یا بعد از آن، در این گروه شغلی مشخص نیست. اين پژوهش به بررسي پوسچر سر در دندان‌پزشكان فاقد درد گردن پرداخته است. تلاش بر این بود با بررسي تغييرات وضعيت سر در دندان‌پزشكان قبل از وقوع درد، به يكي از علل درد گردن احتمالی اين گروه شغلي در آينده پي برده شود. مواد و روش‌ها: در اين مطالعه 41 نفر دندان‌پزشك (گروه آزمون) و 40 نفر غیر دندان‌پزشک (گروه شاهد) انتخاب شدند. بدين‌ منظور از روش استاندارد اندازه‎گیری قوس گردن، جهت بررسی پوسچر سر در دو گروه، بهره گرفته شد. همچنین اطلاعات عمومی مورد نياز از طريق پرسش‌نامه جمع‌آوري گرديد. یافته‌ها: بين ميزان قوس گردن دندان‌پزشكان و افراد گروه شاهد در این پژوهش هيچ اختلافي مشاهده نگرديد (05/0 P >). همچنين قوس گردن بین دو گروه دندان‌پزشك با سابقه‌ كاری 8-5 سال و 12-8 سال نيز با یکدیگراختلافی نداشت (05/0 P >)، تنها تفاوت در ميزان قوس گردن، بين زنان و مردان دندان‌پزشك مشاهده گرديد (05/0> P). بحث: احتمال می‎رود عدم وجود اختلاف در ميزان قوس گردني گروه دندان‎پزشکان و گروه شاهد، به‌ علت عدم وجود درد بوده است.

Title: بررسی مقایسه ای تأثیر سه روش درمانی جریانهای الکتریکی عملکردی، بیوفیدبک و تمرین درمانی بر پارامترهای قدرت عضلانی اندام تحتانی، کینماتیک راه رفتن و بالانس بیماران مبتلا به سکته مغزی

http://jrrs.mui.ac.ir/index.php/jrrs/article/view/117

چکیده مقدمه: سكته مغزی مهم‎ترین عامل در ایجاد ناتوانی و معلولیت در افراد بالغ می‎باشد. اغلب بیماران سكته‎ای، دوباره به طور مستقل می‎توانند راه بروند؛ در حالی که مشكلات حركتی كه در نتیجه اختلال تعادل، ضعف حركتی و كاهش سرعت راه رفتن است، در این افراد ادامه می‎یابد. استفاده از روش‎های درمانی مؤثر جهت بهبود ناتوانی این بیماران ضروری است. مواد و روش‌ها: این پژوهش، از نوع كارآزمایی بالینی غیر تصادفی بود كه در سال 88-1387 در شهر اصفهان انجام گرفت. در این مطالعه، 30 بیمار با میانگین سنی 1/60 سال انتخاب شدند كه حداقل 6 ماه از سكته آن‎ها گذشته بود. بیماران در سه گروه درمانی طی 12 جلسه به صورت یك روز در میان تحت درمان قرار گرفتند. به منظور بررسی تعادل از Berg balance scale، جهت ارزیابی قدرت عضلانی از Manual muscle testing و برای ارزیابی راه رفتن از دستگاه Motion analyzer استفاده شد. اطلاعات حاصل از انجام آزمون‎ها، قبل و بعد از درمان، جمع‎آوری گردید. جهت تجزیه و تحلیل آماری داده‎های به دست آمده، برای مقایسه وضعیت بیماران در مراحل مختلف از آزمون t زوج شده و جهت مقایسه روش های درمانی از آزمون ANOVA موجود در نرم‎افزار SPSS استفاده گردید. یافته‌ها: از نظر بهبود تعادل، قبل و بعد از درمان بین دو گروه جریان‎های الكتریكی عملكردی و تمرین درمانی (01/0 > P) و بین دو گروه جریان‎های الكتریكی عملكردی و بیوفیدبك تفاوت معنی‎داری وجود داشت (01/0 = P)، اما بین دو گروه تمرین درمانی و بیوفیدبك تفاوت معنی‎داری دیده نشد (23/0 = P). از نظر بهبود قدرت عضله كوادریسپس، قبل و بعد از درمان بین دو گروه جریان‎های الكتریكی عملكردی و تمرین درمانی (017/0 = P) و بین دو گروه جریان‎های الكتریكی عملكردی و بیوفیدبك (01/0 = P) تفاوت معنی‎داری وجود داشت، اما بین دو گروه تمرین درمانی و بیوفیدبك تفاوت معنی‎داری دیده نشد (98/0 = P). بین وضعیت راه رفتن بیماران پس از درمان بین سه گروه تنها در طول گام و طول قدم تفاوت معنی‎داری وجود داشت. (017/0 = 2PGST، 017/0 = 2PGSL) بحث: روش بیوفیدبك، مؤثرتر و روش جریان‎های الكتریكی عملكردی كم اثر‎تر از روش‎های ذكر شده در درمان بیماران مبتلا به سكته مغزی بود.

Title: تأثیر بلندی صدا بر خیشومی شدگی واکه‌ها در دانشجویان 18 تا 28 سال طبیعی فارسی زبان

http://jrrs.mui.ac.ir/index.php/jrrs/article/view/118

چکیده مقدمه: خیشومی شدگی یکی از پارامترهای مهم در آسیب‌شناسی تشدید صدا است. صدای افراد عادی نیز به درجاتی خیشومی شده است. به نظر می‌رسد خیشومی شدگی مانند سایر پارامتر‌های صدا، می‌تواند تحت تأثیر فاکتور‌های دیگر همچون بلندی صوت باشد که در ارزیابی آزمایشگاهی قابل اندازه‌گیری است. این پژوهش به منظور بررسی این رابطه در افراد 18 تا 28 ساله عادی فارسی زبان انجام شد. مواد و روش‌ها: در این پژوهش توصیفی- تحلیلی و مقطعی، 65 نفر از دانشجویان دختر و پسر 18 تا 28 ساله فارسی زبان دانشکده علوم توان‌بخشی علوم پزشکی ایران به صورت تصادفی ساده انتخاب شدند و نمونه صدای آن‎ها مورد مطالعه قرار گرفت. میانگین خیشومی شدگی در واکه‌های زبان فارسی با استفاده از نرم‎افزار Nasal view محاسبه شد. داده‌های به دست آمده با استفاده از آمار توصیفی و آنالیز واریانس یک‌ طرفه مورد تجزیه و تحلیل قرار گرفت. یافته‌ها: در زنان و مردان بیشرین اندازه خیشومی شدگی واکه‌ها، در صدای آرام و کمترین خیشومی شدگی، در صدای بلند بود. تحلیل داده‌ها با آزمون‌های آماری نشان داد که خیشومی شدگی واکه‌ها در بلندی صدای آرام، معمولی و بلند صدا تفاوت معنی‌دار داشت (05/0 > P). بحث: در افراد عادی با افزایش بلندی صدا، خیشومی شدگی واکه‌ها کاهش می‌یابد که جنبه‌ای از عملکرد دستگاه طبیعی گفتار است. با توجه به عملکرد دریچه کامی- حلقی و افزایش هوای زیر چاکنای در صداهای بلند و بالا رفتن انقباض عضلانی حلق و کام این یافته‌ها، قابل توجیه است.

Title: آیا خستگي عضلانی بر ثبات عملکردی مردان ورزشكار تاثیر دارد؟

http://jrrs.mui.ac.ir/index.php/jrrs/article/view/122

چکیده مقدمه: خستگی عضلانی طی فعالیت‌های ورزشی پدیده معمولی است که باعث اختلال در عملکرد حرکتی می‌شود. هدف این پژوهش بررسی تأثير خستگي بر ثبات عملکردی مردان ورزشكار بود. مواد و روش‌ها: 42 ورزشکار حرفه‌ای با میانگین سن 2/3 ± 2/25 سال و میانگین وزن 3/8 ± 4/80 کیلوگرم انتخاب شدند و به طور تصادفی در دو گروه شاهد و تجربی قرار گرفتند. در این تحقیق از آزمون RUNING ANAEROBIC SPRINT TEST (RAST) برای ایجاد خستگی، از دستگاه بایودکس (درجه بی‌ثباتی 4) برای اندازه‌گیری ثبات عملکردی و از لاکتومتر جهت اندازه‌گیری لاکتات خون استفاده شد. داده‌های پژوهش با کمک آزمون‌های آماری t مستقل و وابسته مورد تجزیه و تحلیل قرار گرفت (05/0 ≤ α). یافته‌ها: نتایج پژوهش حاضر نشان داد که خستگی عضلانی بر متغیر ثباتی قدامی خلفی تغییر معنی‌داری نداشت (31/0 = P). پس از ایجاد خستگی عضلانی در متغیر ثباتی طرفی (003/0 ≤ P) و محدوده ثبات (002/0 ≤ P) از لحاظ آماری تغییر معنی‌داری مشاهده شد. بحث: با توجه به نتایج به دست آمده، شاید خستگی عضلانی بتواند متغیر ثبات عملکردی ورزشکاران را در جهات طرفی دچار تغییر کند، اما احتمال دارد که در جهت قدامی- خلفی تغییری نداشته باشد. اثبات این مورد نیازمند تحقیقات بیشتری می‌باشد.

Title: ‎راهکارهای اصلاح مکالمه در کودکان طبیعی3 تا 5 ساله شهر اصفهان

http://jrrs.mui.ac.ir/index.php/jrrs/article/view/120

چکیده مقدمه: مهارت‎هاي اصلاح مكالمه، يكي از مهم‎ترين مهارت‎هاي كاربرد شناختي زبان و از دسته مهارت‎هاي مكالمه‎اي مي‎باشد. اهميت اين مهارت‎ها به وي‍ژه در مورد كودكان دچار نقايص زباني از قبيل كودكان كم شنوا يا دچار نقص ويژه زبان كه نياز به اصلاح نقايص ارتباطي دارند، به شدت احساس مي‎شود. اطلاعات آسيب شناسان گفتار و زبان در زمينه چنين مهارتي و شكل طبيعي استفاده از راه‎كارهاي اصلاح مكالمه (تكرار، بازگويي، افزايش و اصلاح سرنخي) بسيار محدود و مبتني بر حدس و گمان مي‎باشد. اين پژوهش به معرفي و مقايسه اين راه‎كارها در كودكان طبيعي 3، 4 و 5 ساله پرداخت. مواد و روش‌ها: در اين پژوهش توصيفي- تحليلي و مقطعي، كاربرد انواع راه‎كارهاي اصلاح مكالمه در 72 نمونه از كودكان دختر و پسر فارسي زبان شهر اصفهان، در سه گروه سني 3، 4 و 5 ساله به كمك دو كتابچه تصويري و ايجاد موقعيت درخواست توضيح، مورد بررسي قرار گرفت. یافته‌ها: راه‎كارهاي بازگويي، اصلاح سرنخي و پاسخ‎هاي نامناسب با سن ارتباط معني‎داري داشتند، ولي در مورد راه‎كارهاي تكرار و افزايش، چنين ارتباط آماري به اثبات نرسيد. در مورد جنس نيز تنها راه‎كار بازگويي در پسران، كاربرد بيشتري نسبت به دختران داشت. بحث: تكرار در هر مقطع سني پركاربردترين راه‎كار بود. استفاده از راه‎كار بازگويي در سنين پايين‎تر، شايع‎تر و با افزايش سن كاهش یافت. استفاده از راه‎كار اصلاح سرنخي نيز با افزايش سن بيشتر شد. اين نتايج با نتايج پژوهش‎هاي خارج از كشور هم‎خواني داشت.

Title: ‎تأثیر ناهنجاری پشت تابدار بر تغییر شكل بخشهای مختلف بدن

http://jrrs.mui.ac.ir/index.php/jrrs/article/view/121

چکیده مقدمه: پژوهش حاضر بررسی تأثیر ناهنجاری پشت‌ تابدار بر تغییر شكل بخش‎های مختلف بدن بود. مواد و روش‌ها: جهت اجرای این پژوهش، تعداد 70 دانشجوی دختر به طور تصادفی ساده انتخاب شدند. برای بررسی وضعیت بدنی آزمودنی‎ها، از صفحه شطرنجی و خط شاقولی استفاده شد. برای اندازه‎گیری شیب لگنی آزمودنی‎ها از شیب‎سنج لگنی، برای اندازه‎گیری میزان لوردوز کمری و کیفوز پشتی از خط‎کش قابل انعطاف استفاده گردید. همچنین زوایای مفاصل زانو، ران و مچ‎ پا با استفاده از گونیامتر اندازه‎گیری شد. از آزمون همبستگی Pearson و آزمون t جهت تجزیه و تحلیل داده‎ها استفاده گردید (05/0 ≥P ). یافته‌ها: نتایج پژوهش نشان داد که میزان شیوع ناهنجاری پشت ‎تابدار بالا می‎باشد (35 درصد). افراد دارای پشت تابدار، میزان تیلت قدامی لگنی و میزان نوسان لگنی بیشتری نسبت به گروه نرمال داشتند. همچنین افراد دارای این ناهنجاری، دارای اکستنشن بیشتری در مفاصل ران و زانوی خود بودند. میزان نوسان لگن با زاویه زانو و ران به ترتیب رابطه معنی‎داری (02/0 = r) و (01/0 = r) داشت، اما این اندازه با زاویه مچ پا رابطه معنی‎داری نشان نداد (06/0 = r).  نتیجه‎گیری: نتایج پژوهش نشان داد که میزان شیوع ناهنجاری پشت تابدار در افراد مورد مطالعه 26-18 سال بالا بود. احتمال ایجاد تغییرات خاص در میزان زوایای لودوز کمری و گردنی، کیفوز سینه‎ای و زوایای ران و زانو این افراد در اثر زمان وجود دارد.

Title: ‎ ( در بيماران اسكيزوفرني تهران(سال86-85 Kohlman پايايي و روايي آزمون مهارتهاي زندگي

http://jrrs.mui.ac.ir/index.php/jrrs/article/view/119

چکیده مقدمه: با توجه به مشكلات بيماران اسكيزوفرني در انجام مهارت‌هاي زندگي،ارزشيابي مهارت‌هاي زندگي در اين بيماران ضروري به نطر مي‌رسد. بر اين اساس، ارزيابي مهارت‌هاي زندگي Kohlman كه در سال 1978 توسط Linda Thomson Kohlman در سياتل ايالت واشنگتن طرح‌ريزي شد، به فارسي برگردانده، پايايي و روايي آن در بيماران اسكيزوفرني تهران بررسي گرديد. مواد و روش‌ها: اين پژوهش به شيوه مقطعي توصيفي- تحليلي انجام شد و پايايي بازآزمایی، پايايي بين ارزيابان، روايي همزمان و روايي سازه آزمون بررسي گردید. به اين منظور 105 بيمار اسكيزوفرني سرپايي، 35 بيمار اسكيزوفرني بستري و 35 نفر از افراد سالم جامعه مورد ارزيابي قرار گرفتند. یافته‎ها: مقايسه نمرات حاصل از ارزيابي دو آزمونگر در 30 بيمار سرپايي (997/0 = r) و نتايج حاصل از تكرار آزمون در 24 بيمار سرپايي (967/0 = r) نشان داد كه آزمون از پايايي مناسب برخوردار است. روايي همزمان از طريق محاسبه همبستگي بين نمره آزمون Kohlman و آزمون مقياس ارزيابي كلي در 35 بيمار بستري تعيين شد (86/0- = r). توانايي تمايز مهارت‌هاي زندگي افراد سالم از بيمار، نشان دهنده روايي سازه آزمون مهارت‌هاي زندگي Kohlman است. بحث: آزمون مهارت‌هاي زندگي Kohlman كه طي اين پژوهش مورد بررسي و بازبيني قرار گرفت، از پايايي و روايي لازم در جمعيت بيماران اسكيزوفرني تهران برخوردار است.

Title: مشاهدات اولیه مقایسه تأثير جملات ساده و پيچيده بر ميزان نارواني گفتار كودكان لكنتي و غيرلكنتي فارسي زبان 6-4 ساله در تکلیف تقلید جملات

http://jrrs.mui.ac.ir/index.php/jrrs/article/view/123

چکیده مقدمه: پژوهش‌هاي متعددي به بررسی ارتباط لكنت با عوامل زبان‎شناختي مختلف پرداخته‌اند؛ به طوری که بعضی از این نتایج نشان می‎دهند که در کودکان لکنتی گفته‌هاي ناروان نسبت به گفته‌هاي روان پيچيد‌ه‌تر هستند. هدف از اين پژوهش، تعيين و مقايسه تأثير جملات ساده و پیچیده بر ميزان نارواني گفتار كودكان لكنتي و غيرلكنتي فارسي زبان در تکلیف تقلید جملات بود. نتايج حاصله شاید بتواند در جهت شناخت بيشتر ماهيت لكنت و نيز يافتن شيوه‌هاي مناسب‌تر براي درمان آن كاربرد داشته باشد. مواد و روش‌ها: اين مطالعه، يك مطالعه‌ توصيفي- تحليلي از نوع مقطعی بود. 10 كودك لكنتي و 10 كودك غيرلكنتي تك زبانه و فارسي زبان 6-4 سال مورد مطالعه قرار گرفتند كه از لحاظ سن و جنس با همديگر مطابقت داشتند. در این پژوهش، دو گروه جمله، شامل 13 جمله ساده و 10 جمله پيچيده، در نظر گرفته شد؛ به طوری که بر طول جملات هر گروه، به ترتیب یک تکواژ افزوده گردد. از هر كودك درخواست ‌شد پس از شنیدن هر جمله، به طور دقیق آن را تكرار كند. ميزان نارواني در هر گفته از تقسيم تعداد كلمات ناروان بر كل كلمات آن گفته محاسبه و به صورت درصد بيان ‌شد. درصد ناروایی جملات ساده و پيچيده در هر گروه و نیز بین دو گروه، با استفاده از نرم‎افزارSPSS  مورد تجزيه و تحليل قرار گرفت. یافته‌ها: در کودکان لکنتی و غیرلکنتی بين جملات ساده و پيچيده از لحاظ ميزان نارواني تفاوت معنی‎داري وجود داشت (05/0 > P). بحث: نتايج اين پژوهش نشان داد که در هر دو گروه، میزان ناروانی در جملات پیچیده بیشتر از جملات ساده بود. هم‌چنین کودکان لکنتی با افزایش پیچیدگی نحوی، دچار میزان ناروانی بیشتری نسبت به کودکان غیرلکنتی ‌شدند.

Title: نيمرخ ناهنجاريهاي بدني (استخواني) ورزشکاران پرورش اندام

http://jrrs.mui.ac.ir/index.php/jrrs/article/view/124

چكيده مقدمه و هدف: ناهنجاريهاي وضعيتي معمولا به دليل استفاده غير استاندارد از بدن حاصل مي شود كه متاسفانه نه تنها باعث بد شكلي بدن بلكه عوارض بسياري نيز به دنبال دارد. هدف از این تحقیق بررسی نوع و میزان ناهنجاری های بدنی در ورزشکاران رشته پرورش اندام بود. مواد و روش ها:  ناهنجاريهاي وضعيتي تعداد 118 نفر از ورزشكاران رشته پرورش اندام به وسیله صفحه شطرنجی و درج در فورم ارزیابی مورد بررسی قرار گرفت . از  مجذورخی  براي آناليز داده ها استفاده شد. نتایج: در كل تعداد 874 ناهنجاري وضعيتي در 118 بدنساز مورد مطالعه مشاهده شد. بطور ميانگين هر ورزشكار حدود 5/7 ناهنجاري داشت. در خصوص نوع ناهنجاري، چرخش داخلی شانه شايعترين نوع ناهنجاری بود (4/10%) و بعد از آن لوردوز کمری (61/9%)، تيلت قدامي لگن (65/8 %) ، شانه افتاده (23/8 %)، کایفوز (78/7%) متداولترين ناهنجاريها بودند. اختلاف معناداری بین میزان ناهنجاری های تنه و سر با اندام فوقانی (01/0>P ) و اندام تحتانی (05/0> P) مشاهده شد، اما بین میزان ناهنجاری های اندام تحتانی و فوقانی تفاوت معناداری مشاهده نشد (05/0> P). بحث و نتیجه گیری: درمجموع مي توان نتيجه گيري كرد كه میزان ناهنجاری ها در ورزشکاران  رشته پرورش اندام بالا می باشد كه بايست در مطالعاتي آتي دلايل بروز اين ناهنجاريها بررسي شود و اين زنگ خطري براي ورزشكاران اين رشته محسوب مي شود، لذا  مربيان بايد تمهيداتي بینديشند كه میزان اين ناهنجاريها را کاهش دهند.
